# Dengue as a growing global health concern

**DOI:** 10.1016/j.eclinm.2024.102975

**Published:** 2024-11-25

**Authors:** eClinicalMedicine

According to WHO's new global dengue surveillance system (launched May, 2024), there have been approximately 13 million reported cases (almost 42,000 of them severe) and over 8700 dengue-related reported deaths worldwide since the beginning of 2024. All WHO regions are affected, with 90 countries having known active transmission. Most cases have been reported in tropical and subtropical areas where the virus is endemic: region of the Americas (12,026,698 cases and 7152 deaths), South-East Asia region (357,808 cases and 1379 deaths) and Western Pacific region (219,441 cases and 154 deaths). Dengue cases have also been reported in non-endemic areas, such as mainland Europe. This recent surge has been preceded by an estimated 85·5% increase in incident cases of dengue worldwide between 1990 and 2019, and a doubling in the number of global cases each year since 2021. On Dec 21, 2023, WHO assigned the highest level of emergency (grade 3) to the global increase in dengue cases and, in June 2024, the WHO Pathogens Prioritization framework selected *Orthoflavivirus denguei* as a priority pathogen in all six WHO regions.

Dengue is a major vector-borne disease transmitted to humans via infected female mosquitoes. Most infected individuals show no or mild symptoms (eg, fever, muscle and joint pains, headache, and nausea), but severe disease can develop in rare cases, characterised by severe plasma leakage, bleeding, and organ impairment. A well-known risk factor for the development of severe dengue is previous infection with a heterologous dengue virus (DENV), but it is thought that multiple mechanisms and factors could also have a role in progression to severe disease. An estimated 4 million cases are estimated to require hospitalisation each year and the median case fatality rate of severe dengue is up to 5%, with severe hepatitis, dengue shock syndrome, altered mental status, diabetes, and a higher pulse rate identified as risk factors for mortality in infected patients.

Dengue transmission is influenced by environmental, socioeconomic, and demographic factors. Climate change, increased human mobility, population growth, urbanisation, and poor water and sanitation provide favourable conditions for dengue transmission. Increasing temperatures are associated with epidemic outbreaks, and modelling data suggest that climate change has expanded the range of *Aedes* vectors and might accelerate their invasion potential to new regions. Dengue is also spread by travel, with most cases of dengue in Europe occurring in people who were infected in other high-risk regions, which can lead to sporadic local transmission when suitable vectors are also established in the area. Unfortunately, many vector-control efforts for sustained management or epidemic mitigation (eg, using chemical insecticides) show unsustainable or limited efficacy, and there is scarce evidence showing that these strategies actually reduce dengue incidence in people. Additionally, long term data on the effectiveness of particular vector-control strategies, such as infection of *A aegypti* mosquitos with *Wolbachia pipientis* bacteria, are still needed.

A crucial component of outbreak responses is diagnosis and surveillance. Assessing the global burden of dengue is challenging due to the large proportion of asymptomatic and mild dengue cases, and a lack of robust detection and reporting systems across endemic regions, a coordinated genomic surveillance network, and accurate, specific, and low-cost rapid diagnostic tests.

In the absence of specific treatments or antivirals for dengue, vaccination is an important strategy to reduce transmission. There are two licensed live-attenuated tetravalent vaccines currently available, CYD-TDV (Dengvaxia; Sanofi Pasteur) and TAK-003 (Qdenga; Takeda Pharmaceuticals). WHO recommends that CYD-TDV be administered as three doses, 6 months apart, to individuals aged 6 years or older who are seropositive for DENV and live in highly endemic areas. CYD-TDV has been shown to confer an 80% lower risk of virologically confirmed dengue (VCD) and VCD hospitalisation in children when compared with unvaccinated controls, but an increased risk of these outcomes has been observed in vaccine recipients who had not been previously exposed to dengue. Unfortunately, the requirement for pre-vaccination screening of serostatus has limited the use of CYD-TDV in national vaccination programmes. In May 2024, WHO announced prequalification of the two-dose TAK-003 vaccine in children aged 6–16 years living in high-transmission settings. The 4.5-year follow-up results of an ongoing multicentre phase 3 trial of the TAK-003 vaccine in dengue-endemic countries showed cumulative vaccine efficacy of around 60% against VCD and 84% against VCD hospitalisation. Efficacy and safety against all four DENV serotypes in children aged 4–16 years previously exposed to dengue was observed, and against DENV-1 and DENV-2 in those previously unexposed. However, the effect of TAK-003 on cases caused by DENV-3 or DENV-4 strains that are severe or require hospitalisation is unknown and being investigated in ongoing work. Other important vaccines in development include the single-dose tetravalent TV003 and TV005 vaccines, which have shown durable immunogenicity (TV005) or efficacy against symptomatic VCD (TV003) in individuals, regardless of previous exposure to dengue. Despite promising advances, long-term data on the efficacy of vaccines are still needed, as well as the identification of a reliable immune correlate of protection.

To reduce the burden of disease and deaths from dengue and other *Aedes-*borne arboviral diseases, WHO launched a global strategic preparedness, readiness, and response plan (SPRP) on Oct 3, 2024. The SPRP aims to understand the current global epidemiological landscape of dengue to inform guidance on preparedness and response strategies, and consists of five key components: emergency coordination; collaborative surveillance; community protection; safe and scalable care; and access to countermeasures. WHO requires US$.55 million to support these actions, which will be implemented until September, 2025. The SPRP outlines clear key performance indicators and targets for each intervention component, as well as how frequently they will be assessed during this timeframe.

Dengue is a huge burden to human health, and the global population at risk is predicted to rise to 63% in 2080. Questions remain about immune correlates of protection, particularly in terms of vaccine efficacy, the effectiveness of vector-control strategies on disease prevention, and the impact of climate change on disease transmission. There is an urgent need to develop and support effective dengue surveillance strategies to understand DENV epidemiology and measure the success of interventions, as well as to foster collaboration between stakeholders to facilitate rapid responses to outbreaks. Moving towards an integrated approach, involving both vector-control strategies and vaccination, is necessary to eliminate dengue as a public health burden.

